# Complexities of the Prodromal Phase of First-Episode Psychosis: A Longitudinal and Phenomenological Diagnostic Approach

**DOI:** 10.1192/j.eurpsy.2025.2049

**Published:** 2025-08-26

**Authors:** A. Guiomar, C. P. Osório, P. M. Martins, P. Macedo, M. de Barros

**Affiliations:** 1 Psychiatry, Unidade Local de Saúde do Tâmega e Sousa, Penafiel, Portugal

## Abstract

**Introduction:**

A 26-year-old man presented with his first-episode psychosis (FEP) following a 15-month period marked by a progressive sense of estrangement from his surroundings, ideas of reference, persistent anxiety, difficulty focusing, and social withdrawal. Two years prior, he began stimulant treatment for suspected attention-deficit/hyperactivity disorder (ADHD), though he discontinued the medication shortly after, as he perceived no improvement. Over the past year, he became increasingly distant from friends and eventually resigned from his job. About three months before hospitalization, he began experiencing first-rank symptoms of schizophrenia. This case will serve as a starting point to discuss the complexities of diagnosing the prodromal phase of FEP.

**Objectives:**

This clinical review aims to examine the phenomenology of the prodromal phase of FEP and address the diagnostic challenges posed by symptom similarities between this phase and neurodevelopmental conditions like ADHD.

**Methods:**

A literature review was conducted using the PubMed database, covering studies from the past 20 years. Studies were selected if they included phenomenological descriptions of the prodromal phase in FEP and/ or examined the impact of neurodevelopmental conditions on the emergence of psychosis.

**Results:**

The review identified several key phenomenological markers characterizing the prodromal phase of FEP, which can aid in distinguishing it from other psychiatric conditions. The prodromal phase of FEP is frequently marked by subtle but progressive alterations in cognition, perception, and affect, including experiences such as derealization-depersonalization, ideas of reference, paranoid ideation, and social withdrawal. Evidence suggests that prodromal symptoms intensify over time, evolving from vague unease to specific disruptions in reality testing. Although ADHD and the prodromal phase of a FEP may share some overlapping characteristics - particularly when symptoms are assessed in a cross-sectional manner - ADHD symptoms are generally regarded as stable traits that persist consistently into adulthood.

**Conclusions:**

This case underscores the need for careful differential diagnosis, especially when evaluating individuals in high-risk age groups for psychosis who present with subtle symptoms that do not clearly fit a single diagnostic category. In such cases, clinicians should avoid premature conclusions and instead adopt a longitudinal and comprehensive approach, considering whether genetic, neurodevelopmental, or social risk factors may be contributing to the presentation. A phenomenological perspective can help clinicians detect subtle yet significant shifts in perception, cognition, and affect, enhancing diagnostic accuracy and enabling timely intervention.

**Disclosure of Interest:**

None Declared

